# Ischuria

**Published:** 1829-06

**Authors:** 


### ISCHURIA.

*A singular Case of Ischuria.* By Charles Hastings, m.d. (Worcester Infirmary.)

On the 9th of April, 1814, M. H., aged twenty-three, was admitted an in-patient of the Worcester Infirmary. She represented herself as having been particularly healthy. Within the last week she had been exposed to cold, whilst the catamenia were flowing abundantly. For the first day or two, she only appeared to suffer from feverish symptoms: soon afterwards, however, the secretion of urine became very deficient, and she had difficulty in passing it.

On the evening of her admission she became much worse, and complained especially of pain and tenderness over the whole of the lower part of the abdomen, and in the loins. There was vomiting, and a disposition to convulsion. The lower part of the abdomen was much distended. The catheter was introduced, and ten ounces of urine were drawn off; after which, the pain was relieved. She was ordered to take a scruple of cathartic extract immediately; and one drachm of sulphate of magnesia, dissolved in camphor mixture, three times a day.

The next morning, the bowels had not been moved. She was afflicted with severe headach, as well as the abdominal pains. She had passed no water, and was delirious during the night.

She was cupped on the back, and had a blister applied, and took cathartic mixture every four hours till the bowels moved freely; after which she went into a warm bath.

The symptoms remained for several days very much in the same state. Delirium usually came on during the night. No urine was passed by the natural effort, but about three ounces were drawn off by the catheter in the course of twenty-four hours. She very frequently vomited, and suffered much from pain, tenderness, and tension of the lower part of the abdomen.

On the evening of the 17th, insensibility came on, for which a blister was applied to the back of the neck; the pulse was sixty. An active aperient was given.

On the 19th, no improvement had taken place, for the vomiting was incessant, and the pain in the abdomen and back was more severe. Pulse eighty. She was bled three days in succession, with some alleviation of the pain, but the abdomen became generally enlarged and very tender; there also ceased to be any urine drawn from the bladder by the catheter. This continued to be the case for five days. The bowels were open. She took saline diuretics without avail.

On the 25th, there was much vomiting, pain, and distention of the abdomen; but she passed a little urine. Pulse eighty. She was bled to eight ounces.

On the 27th, a bloody discharge appeared at the umbilicus, after which the abdominal pain and tension were relieved. She also passed some urine by the urethra. The vomiting was, however, worse than it had previously been.

The bloody discharge from the umbilicus, and the other symptoms, continued very much the same till the 2d of May, when there was a discharge of a urinous appearance and smell from the umbilicus. She had passed no urine by the urethra for three days. The head was very painful, the pupils dilated; pulse fifty-six; bowels costive. Some leeches were applied to the temples, and a blister to the back of the neck; a brisk purge was administered. The catheter was introduced, but no urine found in the bladder.

This discharge of urine from the umbilicus continued till the 5th, when the catamenia appeared, but quickly vanished. The abdomen became less tense and tender; there was not so much vomiting; the bowels were open.

From the 7th to the 9th, there was no discharge of urine from the umbilicus, nor was there any passed by the urethra: as a consequence, the abdomen became much distended, and severe pain followed, with vomiting. The tension was most remarkable at the umbilicus, forming a circumscribed tumor.

On the 10th, in the morning, six ounces of urine were drawn off by the catheter; and, in an hour after, two quarts of urine, of the same appearance, gushed from the umbilicus. This was followed by much relief of the abdominal pains. The discharge of urine from the umbilicus continued for three days, and was accompanied with great improvement of the general symptoms.

The amendment, however, did not last; for the discharge from the umbilicus again ceased, and for three days the vomiting, the headach, the abdominal tension and pain, returned with their former severity.

On the 17th, the catheter was introduced into the bladder, and no urine was found. In an hour after this, two quarts of urine passed from the umbilicus, and soon afterwards great relief was experienced.

From this time to the 25th, there was little variation; but the young woman suffered during that interval very much from vomiting, and daily passed urine from the umbilicus. The catheter was passed every day, and no urine was found, but the bladder contracted strongly on the instrument; sometimes, immediately after the catheter was removed, a discharge of urine would take place by the umbilicus, and once as much as three quarts were thus passed.

On the 26th, for the first time after many days, four ounces of urine were drawn from the bladder. Each succeeding day this quantity was now increased, and the quantity passed by the umbilicus was diminished. There was also a general improvement of the symptoms, with the exception of vomiting: this continued obstinate. All this time the medicine that she took was confined chiefly to the class of purgatives; blisters were also applied to the neck and epigastrium.

The bladder was regularly emptied every day by the catheter for more than a month after this date, during which time the abdominal pain and vomiting subsided, and there was no discharge from the umbilicus. Early in July she began to pass some urine, and the power over the bladder was gradually restored. She was discharged in the middle of July in tolerable health, but still often complained of pain in the pelvic region. She menstruated.

*Observations.*—This curious case of ischuria is well worthy of consideration. The remarkable sympathy observable between the brain, the stomach, and kidneys, is common to all cases of this description, and is so obvious as not to require any further comment.

The very remarkable feature in the case is the occurrence of the urinary discharge from the umbilicus many days after the ischuria had been noticed. Such instances, although rare, are not without parallel in the annals of medicine. Schenck relates two instances of this kind. In the one, a male, the urine was discharged in consequence of an obstruction at the neck of the bladder, "tanquam mictione ex umbilico," for many months, without any detriment to health. In the other, a female, and more resembling the one now related, "cum suppressa per multas dies fuisset urina, tandem per umbilicum urinam profudit."—Schenck, Obs. lib. iii. *de Urina*, p. 489.

The interesting question is to determine in what manner the urine is conveyed to the umbilicus in these instances. The urachus offers itself as a mean by which the discharge may be determined to that part; and it seems probable that, in the case of mechanical obstruction related by Schenck at the neck of the bladder, that a channel of communication was formed by the urachus, between the bladder and the umbilicus. But, in the case we now remark upon, there had been no urine secreted into the bladder long before its appearance at the umbilicus, nor was there for some time after; and the first discharge from the umbilicus was not of an urinary but bloody nature. We must consequently, I think, regard the urinary discharge in this instance as vicarious, and as proceeding probably from the peritoneal surface. This view seems confirmed by the great abdominal distention which took place for some time previous to the discharge from the umbilicus, when it was invariably found, from introducing the catheter, that the bladder was empty, and that it contracted on the instrument.

Some cases of this description have been placed upon record by eminent men worthy of great credit. There is none, perhaps, more deserving of attention than that by PLATERUS, which is thus related by the renowned SENNERTUS: "Puellæ cuidam annos natæ tredecim, cùm aliquando copiosè minxisset, urinam subito suppressam esse, atque tunc aquam serosam ex aure dextra adeo affatim cœpisset effluere, ut una vice mensuræ duæ sœpe emanarint, idque dies aliquot." He then adds, that, on diuretics being administered, the urine was passed freely from the bladder, and the discharge from the ear ceased; but, as soon as the diuretics were discontinued, the discharge again took place from the ear, but was altogether removed by general terebinthinate remedies, and local repellents to the ear. The health did not suffer.—Sennerti Opera, lib. iii. p. 8, § ii. cap. ix.

In our case it was evident that much inflammatory action was going on in the pelvic viscera, previous to and during the discharge of urine from the umbilicus; and there was a considerable sympathy of the general health with the local inflammatory action.

I may further add, as a notice to this case, that the young woman was again admitted into the Infirmary in May, 1827, for paralysis of the lower extremities, from which she recovered by appropriate remedies. The urine for a time was drawn off by the catheter, but there was no return of the former disease.*

* Midland Med. and Surg. Reporter, May 1829.

